# A novel bacteriocin isolated from *Lactobacillus plantarum* W3-2 and its biological characteristics

**DOI:** 10.3389/fnut.2022.1111880

**Published:** 2023-01-10

**Authors:** Zengguang Wang, Yixuan Zhang, Chengcheng Chen, Shichao Fan, Fangming Deng, Lingyan Zhao

**Affiliations:** ^1^College of Food Science and Technology, Hunan Agricultural University, Changsha, China; ^2^Hunan Guotai Foods Co., Ltd., Yueyang, Hunan, China; ^3^Junjie Food Technology Co., Ltd., Shaoyang, China

**Keywords:** bacteriocin, lactic acid bacteria, biological characteristics, separation and purification, antimicrobial activity

## Abstract

In this study, screening bacteriocin-producing strains from 2,000 plant-derived strains by agar well diffusion method was conducted. The corresponding produced bacteriocin was purified and identified by Sephadex gel chromatography, reversed-phase high-performance liquid chromatography (RP-HPLC), and liquid chromatography coupled with tandem mass spectrometry (LC-MS/MS). Meanwhile, the biological characteristics of bacteriocin were investigated. The targeted strain W3-2 was obtained and identified as *Lactobacillus plantarum* by morphological observation and 16S rRNA gene sequence analysis. Correspondingly, a novel bacteriocin (named plantaricin W3-2) produced by *L. plantarum* W3-2 with a molecular weight of 618.26 Da, and an amino acid sequence of AVEEE was separated, purified by Sephadex gel chromatography and RP-HPLC, and identified by LC-MS/MS. Further characteristics analysis displayed that plantaricin W3-2 had good thermal, pH stability, and broad-spectrum antimicrobial ability. In conclusion, plantaricin W3-2 can be used as a new food preservative.

## 1. Introduction

Antimicrobial peptides (AMPs) are a kind of small molecular peptides with antimicrobial activity, which widely exist in animals, plants, and microorganisms (1). Natural antimicrobial peptides have an antimicrobial effect on bacteria, fungi, and viruses (2). Recently, bacteriocin, as a kind of natural antimicrobial peptide, has been extensively studied and become a potential alternative to chemical preservatives. Bacteriocins, which are ribosomally synthesized in prokaryotes, can inhibit or kill phylogenetically related and/or unrelated microorganisms, but they have no antimicrobial effect on bacteriocin-producing strains themselves (3).

A large number of bacteriocin-producing lactic acid bacterias (LABs) have been successfully isolated from Chinese traditional fermented food. Yi Lanhua et al. isolated *Lactobacillus pentosum* DZ35 from salting meat products and obtained bacteriocin pentocin DZ1 and pentocin DZ2, with molecular weights of 4004.03 and 12719.37 Da, respectively (4). Benmouna et al. isolated *Enterococcus* CM9 from camel milk–producing bacteriocin with a molecular weight of 7.6 KDa (5). Ramakrishnan et al. isolated *Lactobacillus rhamnosus* L34 from curds producing bacteriocin with a molecular weight of 5.6 KDa (6). Although a great deal of work has been carried out to study the purification, structure, and characteristics of bacteriocin secreted by LABs, most bacteriocins could not be widely and effectively used in food because of narrow antimicrobial spectrum, poor thermal stability, and high production cost (7). Nisin is the only natural preservative approved by FAO/WHO to be used in food (8). Therefore, novel bacteriocins with a broad antimicrobial spectrum and good thermal stability remain to be explored.

In this study, LAB isolated from the plant-derived strain library with antimicrobial activity were screened and identified. Purification, characterization, and antimicrobial properties of a novel bacteriocin were studied to get a novel bacteriocin and evaluate its potential as a biological preservative.

## 2. Materials and methods

### 2.1. Strains and medium

Indicator strains are as follows: *Escherichia coli* (CGMCC9181), *Staphylococcus aureus* (ATCC6538), *Listeria monoeytogenes* (ATCC19115), *Bacillus subtilis*, *Salmonella*, *Bacillus cereus* [CMCC(B)63301], *Shigella* [CMCC(B)51105], *Micrococcus luteus* [CMCC(B)28001], *Pseudomonas aeruginosa* (ATCC15442), and *Proteusbacillus vulgaris* [CMCC(B)49027].

Test strains: 2,000 test strains used in this study were obtained from the plant-derived strain library in the Food Science and Technology of Hunan Agricultural University.

de Man, Rogosa and Sharpe (MRS) broth: caseinase digest 10.0 g/L, beef paste powder 10.0 g/L, yeast paste powder 4.0 g/L, diammonium hydrogen citrate 2.0 g/L, sodium acetate 5.0 g/L, magnesium sulfate heptahydrate 0.2 g/L, manganese sulfate tetrahydrate 0.05 g/L, dipotassium hydrogen phosphate 2.0 g/L, glucose 20.0 g/L, and tween-80 1.08 g/L.

Nutrient broth: Peptone 10.0 g/L, beef paste powder 3.0 g/L, and sodium chloride 5.0 g/L.

### 2.2. Preparation of cell-free fermentation supernatant (CFS)

Strains were inoculated in MRS broth and cultivated at 37°C for 24 h. CFS was obtained by centrifugation at 4°C and 10,000 r/min for 15 min to remove the bacteria precipitate. The supernatant was collected and filtered through 0.22 μm filter membrane to remove residual bacteria. Samples were stored at 4°C for further analysis (9, 10).

### 2.3. Screening of the antimicrobial potential strains

The bacteriocin-producing potential strains against *E. coli* and *S. aureus* were investigated using the agar well diffusion method according to Bian et al. with a sight modification (11). The pH of CFS was adjusted to 6 using 1 M NaOH (12, 13). Fifteen milliliters of 2% (v/v) plain agar were poured onto a plate to solidify. Three oxford cups were put on it; 100 μl of indicator strain (10^8^ CFU ml^–1^) was inoculated into 200 ml of nutrient broth medium at 50°C. A 20 ml of the mixture was poured onto the agar medium and allowed to solidify. Then, the Oxford cups were removed; 200 μl of CFS (pH = 6) was poured into holes. The plate was placed at 4°C for 4 h and incubated for 12 h at 37°C. The antimicrobial activity was reflected by the growth-free inhibition zones, and the lactic acid solution (pH = 6) was used as the control. The diameters of the inhibition zone were measured with a digital caliper (MNT-150, Shanghai, China) by cross method to determine the antimicrobial activity (14).

### 2.4. Determination of the antimicrobial substances

To clarify whether the antimicrobial activity was derived from the hydrogen peroxide in CFS, 2 mg/ml catalase was added to CFS (pH = 6) at a ratio of 1:1 (v/v) and incubated at 37°C for 2 h. The antimicrobial activity was detected following the description as outlined in Section “Screening of the antimicrobial potential strains” using CFS (pH = 6) as the control, and the CFS with antimicrobial activity was selected for protease test (15, 16). After eliminating the impact of hydrogen peroxide and organic acids, 1 ml of CFS was treated for 2 h at 37°C with 1 mg/ml final concentration of trypsin, protease K, and pepsin (17). The antimicrobial activity was detected following the description as outlined in Section “Screening of the antimicrobial potential strains.” CFS with no protease process was used as the control.

### 2.5. Identification of bacteriocin-producing strains

#### 2.5.1. Morphological characteristics observation

Bacteriocin-producing strains were inoculated into MRS broth and cultured at 37°C for 24 h. After incubation, the colony color and morphology of the strain were observed. A light microscope (CX31, 10 × 100/Oil, Olympus, Tokyo, Japan) was used to observe the colony morphology characteristics of the strain.

#### 2.5.2.16S rRNA gene sequence analysis

Genomic DNA was extracted using bacterial genomic DNA extraction kit (Solarbio, Beijing Solarbio Technology Co., Ltd., Beijing, China). 16S rDNA gene sequence was amplified using the forward primer 5′-AGAGTTTGATCCTGGCTCAG-3′ and the reverse primer 5′-CTACGGCTACCTTGTTACGA-3′. The PCR amplification products were recovered by AxyPrep DNA Gel Recovery Kit (Solarbio, Beijing Solarbio Technology Co., Ltd., Beijing, China) and sequenced by Shanghai Paisano Co., Ltd. (Shanghai, China). 16S rRNA gene sequences were submitted to the NCBI database for homology analysis using the BLAST tool. A phylogenetic tree was constructed by MEGA6 software.

### 2.6. Isolation and purification of bacteriocins

#### 2.6.1. Extraction of bacteriocin

The CFS was concentrated twice using a rotary evaporator at 55°C, mixed with ethyl acetate in a ratio of 1:2, stirred at 100 rpm at 25°C for 2 h, and placed overnight for stratification. The upper organic phase was pooled and evaporated under a vacuum at 55°C using a rotary evaporator (Yarong, SY-5000, Shanghai, China) to obtain the crude bacteriocin extract (18). Samples were stored at 4°C for further analysis.

#### 2.6.2. Sephadex gel chromatography

In all, 2 ml of crude bacteriocin extract was initially filtered with 0.22 μm filter membrane, then samples were loaded in Sephadex G-25 gel chromatography column (Henghuibio, 1.5 cm × 80 cm, Beijing, China) to eliminate impurities, The samples were eluted with sterile water (pH5.5) with a linear gradient at a flow rate of 0.5 ml/min and measured by UV detector at 280 nm. The fractions were collected according to an automatic collection device (19). Using *S. aureus* as an indicator strain, the antimicrobial activity of each fraction was detected by agar well diffusion method.

The fractions with antimicrobial effect were pooled, concentrated, dried, and dissolved in PBS (pH 5.5) as well as loaded onto the Sephadex LH-20 gel chromatography column (Henghuibio, 1.5 cm × 80 cm, Beijing, China) and eluted by 80% methanol at a flow rate of 0.25 ml/min with a UV detector at 280 nm. Each of fractions was collected, and antimicrobial activity was detected (17). The active fractions with antimicrobial activity were pooled for full-wavelength scanning, with the maximum absorption peak as the detection wavelength for the next purification.

#### 2.6.3. RP-HPLC purification

Fractions with antimicrobial effect collected from the Sephadex LH-20 gel chromatography column (Henghuibio, 1.5 cm × 80 cm, Beijing, China) were filtered with 0.22-μm filter membrane and further purified by RP-HPLC (SCG100-V2, Suzhou Sepure Instruments Co., Ltd., Suzhou, China) (20). The purification conditions were as follows: C18 column (Phenomenex Jupiter C18, 4.6 mm × 250 mm, Phenomenex, USA), mobile phase A comprised 85% water and 15% acetonitrile (containing 0.07% trifluoroacetic acid), gradient: 100% A within 35 min, flow rate at 1 ml/min, the volume of samples loaded into the system was 500 μl. UV detector was set at 220 nm (21). A component of each peak was collected, concentrated by freezing, and then, dissolved in PBS (pH 5.5). Antimicrobial activity was detected by agar well diffusion method using *S. aureus* as an indicator strain.

#### 2.6.4. Identification of bacteriocin

Nano liquid chromatography coupled with tandem mass spectrometry analysis was performed to determine the molecular mass and amino acid sequence of the purified bacteriocin. The bacteriocin purified by RP-HPLC (SCG100-V2, Suzhou Sepure Instruments Co., Ltd., Suzhou, China) was reduced by 10 mM DL-dithiothreitol (Sigma-Aldrich, St. Louis, MO, USA) at 56°C for 1 h and alkylated by 50 mM iodoacetamide (Sigma-Aldrich, St. Louis, MO, USA) at room temperature in dark for 40 min. Lyophilize the extracted bacteriocin to near dryness. Resuspend peptides in 20 μl of 0.1% formic acid (Sigma-Aldrich, St. Louis, MO, USA) before LC-MS/MS analysis. The analysis of bacteriocin was conducted by nano LC-MS/MS in an Easy-nLC 1200 system (Thermo Fisher Scientific, Waltham, MA, USA) coupled with a Q Exactive™ Hybrid Quadrupole-Orbitrap™ Mass Spectrometer (Thermo Fisher Scientific, Waltham, MA, USA) with an electrospray nanospray source. An in-house built reverse-phase nanocolumn (150 μm × 15 cm, 1.9 μm, 100 Å, Dr. Maisch GmbH, Germany) packed with Acclaim PepMap was used. Mobile phase A consisted of 0.1% formic acid in water, mobile phase B comprised 20% of 0.1% formic acid in water- 80% acetonitrile. Liquid chromatography linear gradient: from 4 to 8% B for 2 min, from 8 to 28% B for 43 min, from 28 to 40% B for 10 min, from 40 to 95% B for 1 min, and from 95 to 95% B for 10 min. Total flow rate is at 600 nL/min. A first-order mass spectrometry spectrum was obtained in a full-scan positive ion mode of m/z 300–1800. The second-order mass spectrometry data were collected using collision-induced dissociation to capture the *b* and *y* ions (22). The data acquisition and analysis were conducted by PEAKS Studio 8.5 software.

### 2.7. Thermal stability of plantaricin W3-2

Plantaricin W3-2 was heated at 60, 70, 80, 90, 100, and 121°C for 10 min, then cooled to room temperature. The antimicrobial activity was detected by agar well diffusion method, using unheated plantaricin W3-2 as the control (23).

### 2.8. pH stability of plantaricin W3-2

pH value of plantaricin W3-2 was adjusted to 2, 3, 4, 5, 6, 7, 8, 9, 10, 11, and 12 with 1 mol/L HCL and 1 mol/L NaOH, respectively, and heated at 37°C for 2 h by water bath, then pH value was adjusted back to 6. The antimicrobial activity was detected by agar well diffusion method, using untreated plantaricin W3-2 as the control (24, 25).

### 2.9. Antimicrobial spectrum of plantaricin W3-2

The antimicrobial spectrum of plantaricin W3-2 was studied using the agar well diffusion method. *E. coli* (CGMCC9181), *S. aureus* (ATCC6538), *L. monoeytogenes* (ATCC19115), *B. subtilis*, *Salmonella*, *B. cereus* [CMCC(B)63301], *Shigella* [CMCC(B)51105], *M. luteus* [CMCC(B)28001], *P. aeruginosa* (ATCC15442), and *P. vulgaris* [CMCC(B)49027] were used as indicator strains.

### 2.10. Statistical analysis

The study was carried out using three experimental replicates. Results are presented as mean ± SD. Analysis of variance (ANOVA) was used, and the means were separated at a significance level of *p* < 0.05. The data were analyzed and processed using Excel, IBM SPSS Statistics 26, and Origin 2019 software.

## 3. Results and discussion

### 3.1. Screening and identification of bacteriocin-producing lactic acid bacteria

#### 3.1.1. Screening bacteriocin-producing lactic acid bacteria

**FIGURE 1:**
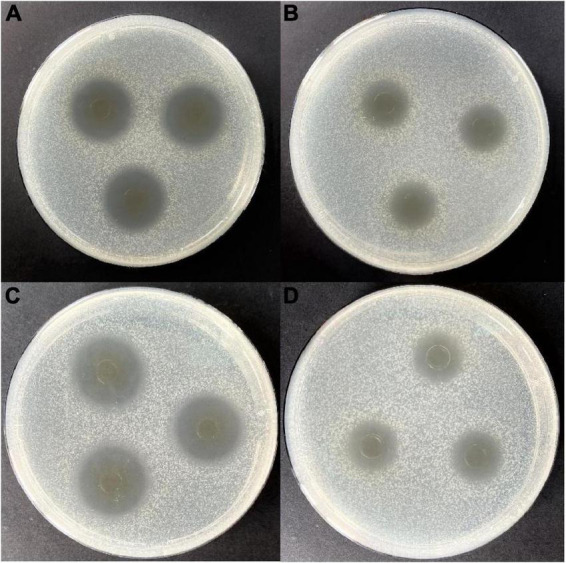
Antimicrobial activity of the cell-free fermentation supernatant (CFS) of strain W3-2 against *Staphylococcus aureus* and *Escherichia coli*. **(A)** Before eliminating the contribution of hydrogen peroxide to the antimicrobial activity on *S. aureus*. **(B)** After eliminating the contribution of hydrogen peroxide to the antimicrobial activity on *S. aureus*. **(C)** Before eliminating the contribution of hydrogen peroxide to the antimicrobial activity on *E. coli*. **(D)** After eliminating the contribution of hydrogen peroxide to the antimicrobial activity on *E. coli*.

Lactic acid bacteria can produce organic acids and hydrogen peroxide, which can inhibit the growth of other strains (26, 27). Both of them have been reported to act on the cytoplasmic membrane by neutralizing its electrochemical potential and increasing its permeability, resulting in bacteriostasis and ultimately death of susceptible bacteria (28). In this study, isolating and screening bacteriocin-producing strains from 2,000 plant-derived strains by agar well diffusion method with *E. coli* and *S. aureus* as indicator strains were conducted. Results indicated that 25 strains were initially isolated after eliminating the contribution of organic acid (Table 1). Further elimination of the contribution of hydrogen peroxide, only strain W3-2 exhibited the strongest antimicrobial activity with the diameters of the inhibition zone against *E. coli* and *S. aureus* exceeded 16 mm (Figure 1). Therefore, strain W3-2 was selected for subsequent tests.

**TABLE 1:** Strains retained antimicrobial activity against *Escherichia coli* and *Staphylococcus aureus* after eliminating the influence of organic acid.

Strain number	Strain source	Mycelial morphology	Diameter of inhibition zone (mm)	
			*E. coli*	*S. aureus*
Lactic acid (pH6)	–	–	–	–
17	Fermented pepper	Bacilli	15.39 ± 0.14^cd^	12.52 ± 0.04^L^
38	Fermented pepper	Bacilli	16.08 ± 0.24^b^	16.34 ± 0.06^C^
3-3	Fermented mustard	Bacilli	13.75 ± 0.08^g^	12.43 ± 0.04^L^
3-20	Fermented mustard	Bacilli	14.10 ± 0.16^f^	15.94 ± 0.13^D^
Z3-10	Fermented pepper	Bacilli	11.19 ± 0.04^m^	12.82 ± 0.08^K^
Z3-19	Fermented pepper	Bacilli	12.13 ± 0.11^K^	11.69 ± 0.04^N^
Z3-27	Fermented pepper	Bacilli	13.20 ± 0.02^i^	13.06 ± 0.08^J^
Z3-35	Fermented pepper	Bacilli	16.06 ± 0.17^b^	15.97 ± 0.08^D^
Z3-37	Fermented pepper	Bacilli	11.98 ± 0.06^K^	11.26 ± 0.07^O^
Z3-38	Fermented pepper	Bacilli	13.77 ± 0.09^g^	12.76 ± 0.07^K^
C1-4	Fermented radish	Coccus	14.01 ± 0.12^f^	16.77 ± 0.08^AB^
C1-5	Fermented radish	Coccus	15.21 ± 0.11^D^	15.17 ± 0.08^f^
C1-10	Fermented radish	Coccus	12.76 ± 0.09^J^	11.17 ± 0.04^OP^
C1-13	Fermented radish	Coccus	12.02 ± 0.17^K^	11.09 ± 0.06^P^
C1-20	Fermented radish	Coccus	15.54 ± 0.05^C^	11.65 ± 0.06^N^
C1-24	Fermented radish	Coccus	15.37 ± 0.08^cd^	13.03 ± 0.08^J^
C2-2	Fermented radish	Coccus	11.66 ± 0.07^L^	11.84 ± 0.07^m^
C2-4	Fermented radish	Coccus	13.36 ± 0.07^hi^	11.95 ± 0.07^m^
W3-2	Fermented pepper	Bacilli	17.54 ± 0.06^a^	16.68 ± 0.04^b^
X13-5	Fermented pepper	Coccus	13.55 ± 0.10^gh^	16.89 ± 0.06^a^
X13-6	Fermented pepper	Coccus	13.36 ± 0.07^hi^	13.72 ± 0.04^i^
X13-7	Fermented pepper	Bacilli	15.52 ± 0.12^C^	14.83 ± 0.06^g^
X13-11	Fermented pepper	Bacilli	14.43 ± 0.04^e^	14.19 ± 0.04^H^
Z1-2	Fermented mustard	Bacilli	15.21 ± 0.04^D^	15.34 ± 0.05^e^
Z3-27	Fermented mustard	Bacilli	13.16 ± 0.07^i^	13.01 ± 0.10^J^

“–” Means no inhibition. Values represent means of three independent replicates ± SD. Different letters within a column indicate statistically significant differences between the means (*p* < 0.05).

In order to confirm the antimicrobial active substance is bacteriocin, the CFS of strain W3-2 was treated with trypsin, pepsin, and proteinase K, respectively. As shown in Table 2, results displayed that the diameters of the inhibition zone against *S. aureus* decreased from 16.68 ± 0.04 to 10.25 ± 0.18 mm, 10.55 ± 0.24 mm, and 13.22 ± 0.15 mm after being treated with trypsin, pepsin, and proteinase K, respectively. These results indicated that bacteriocin was the main antimicrobial substance in CFS of W3-2. Bacteriocin can damage the structure of cell membrane, making the cell membrane form pores, leading to cell rupture and the outflow of small molecules, such as potassium ions, magnesium ions, phosphorus ions, amino acids, and ATP (29). The low level of ATP and ion deficiency in cells result in the inhibition of the synthesis of DNA, RNA, proteins, and polysaccharides, ultimately leading to cell death (30). Bacteriocin BM1122 led to the separation of the plasma wall of *S. aureus* and the formation of pores in *E. coli* and could inhibit the formation of biofilm, disrupt the normal cell cycle, thus damage the integrity of cell membrane (31). In addition, bacteriocin can also induce cell death by changing the permeability of cell membrane or the activity of intracellular enzymes, inhibiting the germination of spores and the respiration of sensitive bacteria interfering with the normal metabolism of nucleic acids, proteins, and other substances in cells (32).

**TABLE 2:** The diameters of the inhibition zone of strain W3-2 against *Staphylococcus aureus* after protease treatment.

Treatment	Diameter of inhibition zone (mm)
CK	16.68 ± 0.04
Trypsin	10.25 ± 0.18
Pepsin	10.55 ± 0.24
Proteinase K	13.22 ± 0.15

#### 3.1.2. Identification of strain W3-2

**FIGURE 2:**
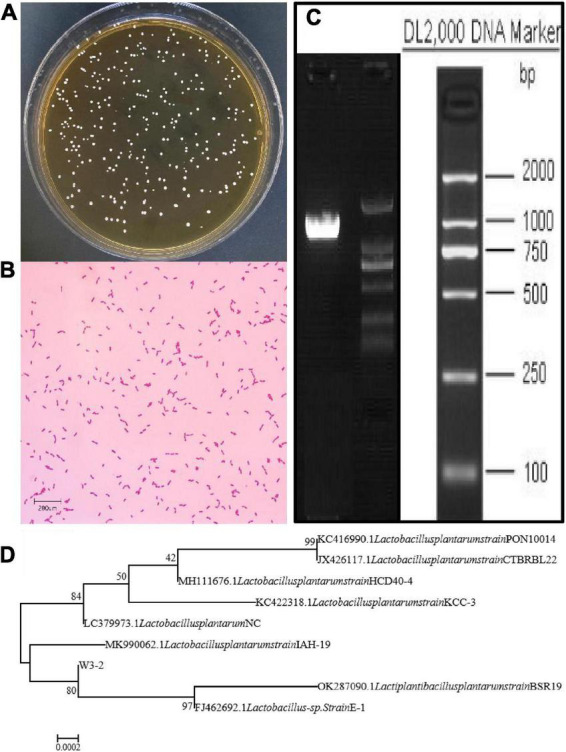
Identification of strain W3-2. **(A)** Colony morphology of strain W3-2. **(B)** Microscopy feature of strain W3-2 after Gram staining (10 × 100/Oil). **(C)** Agarose gel electrophoresis of PCR amplification products of strain W3-2. **(D)** Phylogenetic tree of strain W3-2 based on 16S rRNA sequence.

Strain W3-2 was further identified by colony morphology and 16S rRNA gene sequence analysis. As shown in Figure 2A, the colonies of strain W3-2 were medium-sized, raised, creamy white, rounded, and with neat edges. It is a gram-positive strain with single or arranged in chains, rod-shaped, and spore-free (Figure 2B). The strain W3-2 was amplified by PCR, and the gel electrophoresis results of the PCR are shown in Figure 2C. Results showed that the 16S rRNA gene sequence length of strain W3-2 was 1,466 bp, and the homology with *Lactobacillus plantarum* was more than 99.86% (Figure 2D). Combined morphological characteristics with 16S rRNA gene sequence analysis, strain W3-2 was identified as *L. plantarum*. Swapnil et al. isolated a bacteriocin-producing *L. plantarum* from the colon part of honey bee and the rectum region of stomach (33). Mills et al. isolated *L. plantarum* from handmade cheese that can inhibit the growth of *Listeria* (34).

### 3.2. Purification and identification of the bacterin

#### 3.2.1. Sephadex gel chromatography and RP-HPLC purification

**FIGURE 3:**
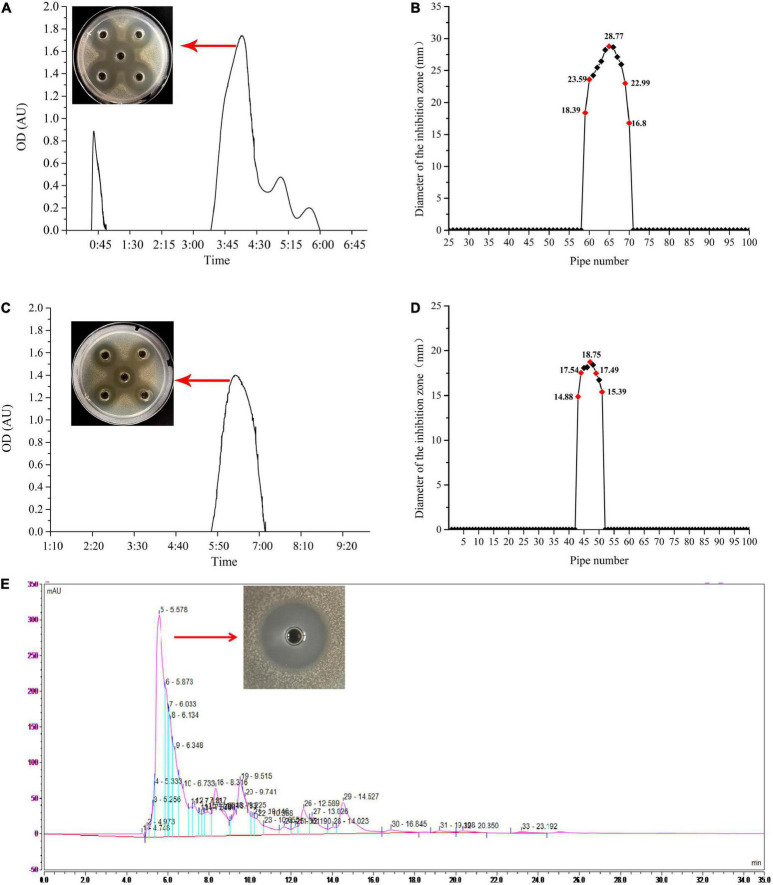
The process of purification of the bacteriocin produced by strain W3-2. **(A)** Sephadex G-25 gel profile. **(B)** Sephadex G-25 elution curve. **(C)** Sephadex LH-20 gel profile. **(D)** Sephadex LH-20 elution curve. **(E)** Reversed-phase high-performance liquid chromatography (RP-HPLC) purification profile.

The ethyl acetate extracted bacteriocin produced by *L. plantarum* W3-2 was purified by gel filtration chromatography. As shown in Figure 3A, there were four elution peaks in the purification process of Sephadex G-25. It was proved that only the second elution peak had antimicrobial activity against *S. aureus*, while the other three elution peaks had no inhibitory effect. The eluent of tubes 59–70 was collected and concentrated (Figure 3B). After further purification by Sephadex LH-20 (Figure 3C), a single elution peak with similar antimicrobial activity was appeared. The eluent corresponding to the 44th–51th tubes was collected for subsequent purification (Figure 3D). There were five absorption peaks after being purified by RP-HPLC (Figure 3E). Except for the one at 5.578 min, the other absorption peaks had no antimicrobial effect. Therefore, eluent at peak time of 5.578 min was collected for further analysis.

#### 3.2.2. Identification of bacteriocin structure by LC-MS/MS

**FIGURE 4:**
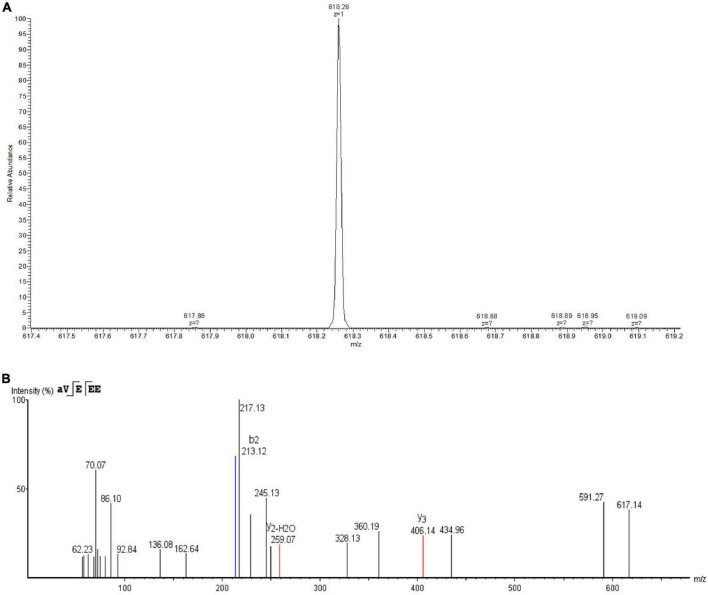
Identification of plantaricin W3-2 by liquid chromatography coupled with tandem mass spectrometry (LC-MS/MS). **(A)** Primary mass spectrometry of plantaricin W3-2. **(B)** Secondary mass spectrometry of plantaricin W3-2.

Eluent at a retention time of 5.578 min was collected and identified by LC-MS/MS. As shown in Figure 4A, the primary mass spectrometry displayed that the molecular weight of the bacteriocin was 618.26 Da and named as plantaricin W3-2. The primary mass spectrum peak was further analyzed by *De novo*, and the secondary mass spectrum was finally obtained. As shown in Figure 4B, the entire amino acid sequence was detected as AVEEE. Plantaricin W3-2 is a polypeptide composed of five amino acids with antimicrobial activity. Bacteriocins, with the same molecular weight and amino acid sequence, have not been reported in the present study.

Most of the molecular weights of bacteriocins reported at present are between 1 and 10 KDa. For example, plantaricin SLG1 produced by *L. plantarum* SLG1 isolated from yak cheese has a molecular weight of 1083.25 Da (35); plantaricin JY22 produced by *L. plantarum* JY22 isolated from the intestine of golden carp has a molecular weight of 4.1 KDa (36); and plantaricin H5 produced by *L. plantarum* H5 isolated from the intestinal flora of sturgeon has a molecular weight of 3.0 KDa (37). Plantaricin W3-2 has a molecular weight of 618.26 Da, which belongs to the small molecular weight bacteriocin. Similarly, the molecular weight of the plantarum C010 is 260.1161 Da produced by *L. plantarum* C010 isolated from cow manure (17).

### 3.3. Biological characteristic of plantaricin W3-2

#### 3.3.1. Thermal stability of plantaricin W3-2

**FIGURE 5:**
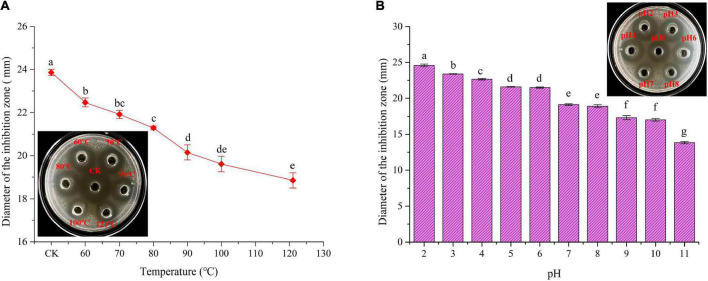
Thermal and pH stability of plantaricin W3-2. **(A)** Thermal stability of plantaricin W3-2, inserted: picture was corresponding inhibition zone, CK was the control group. **(B)** pH stability of plantaricin W3-2. Different letters indicate statistically significant differences between the means (*p* < 0.05).

Thermal stability of plantaricin W3-2 was envaulted. As depicted in Figure 5A, compared to control group, no significant difference in antimicrobial effect on *S. aureus* by plantaricin W3-2 was observed after exposure to 60, 70, and 80°C for 10 min. After being treated at 100°C, 82.2% antimicrobial activity was retained. Further, the increase in temperature to 121°C, and 73.4% antimicrobial activity was kept. Those results elucidated that plantaricin W3-2 had good thermal stability. The reason may be ascribed to small hydrophobic proteins, almost without tertiary structure, with strong hydrophobic region and stable cross-linking structure. The good thermal stability of the plantaricin W3-2 is similar to the bacteriocin produced by *L. plantarum* SLG1 isolated from yak cheese, which exhibits good antimicrobial activity even after being heated at 100°C for 20 min (35).

#### 3.3.2. pH stability of plantaricin W3-2

Results of pH stability of plantaricin W3-2 are shown in Figure 5B, and the antimicrobial activity of plantaricin W3-2 was significantly affected by the change in pH. It was enhanced by acidic environment, while inhabited by basic environment. The antimicrobial activity on *S. aureus* was the largest at pH 2, with diameter of 24.6 mm. The inhibition zone was gradually decreased with the increase in pH and retained 64.3% antimicrobial activity at pH 11. These results proved that plantaricin W3-2 not only had a good antimicrobial effect in acidic environment but also retained its function in a basic environment.

#### 3.3.3. Antimicrobial spectrum of plantaricin W3-2

Ten kinds of common pathogenic bacteria in food were used as indicator strains to test the antimicrobial effect of plantaricin W3-2. Results showed that the diameters of the inhibition zone of plantaricin W3-2 against *S. aureus*, *Listeria monocytogenes*, and *B. cereus* reached 28 mm. For *B. subtilis*, *M. luteus, E. coli*, *P. aeruginosa*, *Salmonella*, *Shigella*, *Proteus*, and other indicator strains, the diameter of the inhibition zone of plantaricin W3-2 was around 21 mm (Table 3). These results demonstrated that plantaricin W3-2 had broad-spectrum antimicrobial activity.

**TABLE 3:** The diameters of the inhibition zone of plantaricin W3-2 against various indicator strains.

Strain properties	Indicator strains	Source	Diameter of inhibition zone (mm)
Gram-positive bacteria	*Staphylococcus aureus*	ATCC6538	29.14 ± 0.12
	*Listeria monocytogenes*	ATCC19115	28.88 ± 0.15
	*Bacillus subtilis*	Lab preservation	21.02 ± 0.22
	*Bacillus cereus*	CMCC(B)63301	28.79 ± 0.15
	*Micrococcus luteus*	CMCC(B)28001	21.80 ± 0.05
Gram-negative bacteria	*Pseudomonas aeruginosa*	ATCC15442	26.91 ± 0.18
	*Escherichia coli*	CMCC9181	27.32 ± 0.14
	*Salmonella*	Lab preservation	21.70 ± 0.42
	*Shigella*	CMCC(B)51105	22.29 ± 0.06
	*Proteus mirabilis*	CMCC(B)49027	23.47 ± 0.15

## 4. Conclusion

In conclusion, *L. plantarum* W3-2 was screened from 2,000 strains in the plant-derived strain library in our laboratory, and corresponding plantaricin W3-2 with 618.26 Da and AVEEE amino acid sequence were obtained. Further, characteristics analysis displayed that plantaricin W3-2 had good thermal, pH stability, and broad-spectrum antimicrobial ability. Safety, application, and antimicrobial mechanism against foodborne pathogens of the plantaricin W3-2 will be investigated in our further studies.

## Data availability statement

The original contributions presented in this study are included in the article/supplementary material, further inquiries can be directed to the corresponding authors.

## Author contributions

FD and LZ designed the work. ZW performed the experimental work and prepared the initial draft of the manuscript. CC and SF critically revised the manuscript. All authors contributed to the article and approved the submitted version.
